# Enhanced pro-apoptotic activity of rituximab through IBTK silencing in non-Hodgkin lymphoma B-cells

**DOI:** 10.3389/fonc.2024.1339584

**Published:** 2024-02-02

**Authors:** Eleonora Vecchio, Rossana Marino, Selena Mimmi, Camilla Canale, Carmen Caiazza, Alessandro Arcucci, Maria Rosaria Ruocco, Marco Schiavone, Gianluca Santamaria, Camillo Palmieri, Enrico Iaccino, Massimo Mallardo, Ileana Quinto, Giuseppe Fiume

**Affiliations:** ^1^ Department of Experimental and Clinical Medicine, University Magna Græcia of Catanzaro, Catanzaro, Italy; ^2^ Department of Molecular Medicine and Medical Biotechnology, University of Naples “Federico II”, Naples, Italy; ^3^ Department of Public Health, University of Naples “Federico II”, Naples, Italy; ^4^ Department of Molecular and Translational Medicine, University of Brescia, Brescia, Italy

**Keywords:** rituximab, non-Hodgkin’s lymphoma, MYC, IBTK, apoptosis

## Abstract

Rituximab is a commonly used chemotherapeutic drug for patients with aggressive lymphomas, such as non-Hodgkin’s lymphoma (NHL). Currently, the combination of Rituximab and chemotherapy (R-CHOP) stands as the most prevalent first-line therapy for NHL. Nevertheless, the development of new therapeutic approaches remains imperative. An increasing body of evidence highlights a novel role for IBTK in tumorigenesis and cancer growth. In this study, we aim to broaden our understanding of IBTK’s function in B-lymphoma, with a particular focus on its impact on the expression of the oncogene MYC. Here, we assessed the effects of combining Rituximab with IBTK silencing on cell viability through cell cycle analysis and Annexin V assays *in vitro*. Furthermore, we leveraged the transplantability of *Eμ-myc* lymphomas to investigate whether the inhibition of IBTK could elicit anti-tumor effects in the treatment of lymphomas *in vivo*. Our data suggests that IBTK silencing may serve as an effective anti-tumor agent for aggressive B-Lymphomas, underscoring its role in promoting apoptosis when used in combination with Rituximab, both in *in vitro* and *in vivo* settings.

## 1 Introduction

Mounting evidence supports a novel role for IBTK in cell survival and tumor growth. IBTK exerts pleiotropic effects, impacting multiple pathways, including protein turnover as a substrate receptor of the Cullin 3 Ubiquitin ligase complex (CRL3^IBTK^) (1), and RNA metabolism, where it modifies RNA splicing in a cell-type-specific manner (2). Recent emerging research has further confirmed that silencing IBTK has the potential to induce apoptosis in hematological malignancies. In chronic lymphocytic leukemia (CLL), the overexpression of IBTK has been correlated with disease progression and proved essential for B cell survival under stress induced by chemotherapeutic agents (3). In the context of MYC-driven lymphomagenesis, the loss of IBTK primarily induces B-cell apoptosis and delays tumor onset (4). Previously, our research has demonstrated that IBTK haploinsufficiency also influences the tumor microenvironment in a mouse model of MYC-driven B-cell lymphoma (5). Recently, we demonstrated that silencing IBTK reduces cell viability and increases apoptosis in malignant B cells. Furthermore, our study revealed that IBTK upregulates the oncogene MYC expression, resulting in decreased survival of malignant B cells. This effect is mediated through the promotion of GSK3β ubiquitylation and proteasomal degradation, which occurs via the β-catenin axis (6). As a result, considerable attention has been directed toward exploring the potential therapeutic value of IBTK in aggressive B lymphomas. However, it’s worth noting that the impact of IBTK on the growth of MYC-driven B lymphomas had not been previously reported.

The tumorigenic potential of MYC often coincides with genetic and epigenetic alterations in hematological malignancies. Lymphomas encompass a highly diverse group of neoplasms that arise from the clonal expansion of B cells, T cells, or natural killer (NK) cells (7). Non-Hodgkin lymphoma (NHL) accounts for 90% of these cases. In Western countries, the most prevalent aggressive NHL subtypes include diffuse large B-cell lymphoma (DLBCL), Mantle Cell lymphoma (MCL), and Burkitt lymphoma. Regarding treatment options, Rituximab, in combination with chemotherapy (cyclophosphamide, doxorubicin, vincristine, and prednisone) or R-CHOP (8), has become the standard of care for adults with B-cell cancers, including patients with diffuse large B-cell lymphoma and Burkitt’s lymphoma (8). However, the emergence of rituximab resistance has become a significant challenge in the treatment of NHL patients (9), underscoring the need for the development of new therapeutic regimens. In this current study, our aim was to investigate the impact of combining Rituximab treatment with IBTK silencing on the growth of MYC-driven B lymphoma. We sought to enhance the effectiveness of anti-CD20 therapy in NHL.

## 2 Materials and methods

### 2.1 Mice

*Eμ-myc* transgenic mice (TgN(IghMyc)22Bri/J) were obtained from The Jackson Laboratory (Bar Harbor, Maine; USA). *Ibtk* ^−/−^ were obtained as previously described (4). Both Eμ-myc transgenic mice and *Ibtk^−/−^* mice were congenic with C57BL/6 J mice. Eμ-myc trans-genic mice were crossed with *Ibtk^−/−^* or *Ibtk^+/−^* mice to generate *Ibtk^+/+^* Eμ-myc and *Ibtk^−/−^ Eμ-myc* littermates. The Eμ-myc transgene was detected by genomic PCR amplification of 600-bp product as described (10), while genotyping for *Ibtk* and *βgeo* genes was performed as previously described (4). Mice were monitored daily for signs of morbidity and tumor development. Moribund mice and mice with obvious tumors were sacrificed, and single-cell suspensions were obtained from tumor tissues and frozen in 10% DMSO for *in vivo* treatment.

### 2.2 Generation of *Eμ-myc* lymphoma in C57BL/6 mice and treatment

To transplant *Eμ-myc* lymphomas to C57BL/6 mice, we thawed, washed, and counted viably frozen *Eμ-myc* lymphoma cells deriving from *Ibtk^+/+^Eµ-myc* and *Ibtk^-/-^Eµ-myc* mice. We suspended the lymphoma cells in RPMI media (GIBCO) and injected 1×10^6^ lymphoma cells by the subcutaneous (s.c.) route into C57BL/6J mice, as previously described (11, 12). We monitored recipient mice daily, and as soon as each tumor volume reached about 100 mm^3^, the mice were randomly assigned to two groups (five mice per group): Vehicle, Rituximab 10 mg/kg (clinical formulation; Roche Diagnostics, Basel-Switzerland). Mice were treated by intraperitoneal injection of Vehicle or Rituximab (11, 12). The tumor volumes were determined by measuring length (L) and width (W) and then calculating volume (V = Length x Width^2^/2) at the indicated time points. At the end of treatment, mice were sacrificed, and the tumors were removed and weighed.

### 2.3 Cells, plasmids, lentivirus, antibodies

HEK293T, in addition to the Burkitt lymphoma cell lines Ramos, Raji, and Daudi, were purchased from Sigma‐Aldrich. It is noteworthy that Ramos cells are EBV-negative, whereas both Raji and Daudi are EBV-positive cell lines. Ramos, Raji and Daudi cells were grown in RPMI (Thermo Fisher Scientific, Waltham, MA, USA). HEK293T cells were grown in Dulbecco’s Modified Eagle Medium (DMEM; Thermo Fisher Scientific, Waltham, MA, USA). Cell culture media were supplemented with 10% fetal bovine serum (FBS), 2 mM L‐glutamine, 1 mM Na‐pyruvate, 50 mM 2βmercaptoethanol, 100 U/mL penicillin, and 100 μg/mL streptomycin; all reagents were purchased from Thermo Fisher Scientific. The plasmids pCMV6‐IBtkα‐FLAG and pCMV6 were from OriGene Technologies, Inc. (Rockville, MD, USA). The lentiviral constructs expressing the short hairpin RNA against IBtkα (shIBTK) or control short hairpin RNA (shCNTL) (TRCN0000082575 and SHC002, respectively) were from MISSION® (Sigma‐Aldrich, St. Louis, MO, USA).

### 2.4 Cells transfection and transduction

HEK293T cells were transfected with plasmids using Lipofectamine 2000 (Thermo Fisher Scientific), according to the manufacturer’s protocol. Lentiviral particles were produced by transfection of HEK 293T cells, as previously described (6). Briefly, HEK293Tcells (1 × 10^6^) were transfected with pCMV‐dR8.91 (5 μg) and pCMV‐VSVG (5 μg) together with shIBTK (10 μg) or shCNTL (10 μg); 48 h post‐transfection, cell supernatant was collected, filtered through 0.22 μm sterile filter, and used for spinoculation in the presence of 8 μg/mL polybrene. For IBtkα silencing, Ramos cells (3 × 10^6^) were transduced with lentiviral particles (500 ng of p24) expressing shIBTK or shCNTL. Twenty‐four hours later, transduced cells were subjected to selection with puromycin (1.5 μg/mL and 0.2 μg/mL, respectively) for 48 h. When required, cells were treated with the RITUXIMAB (Roche Di-agnostics; stock solution 10mg/mL).

### 2.5 Apoptosis and cell cycle analysis

Annexin V‐based apoptotic assay was performed as previously described (4). Briefly, Ramos, Raji, and Daudi cells (1 × 10^6^) were stained with FITC‐conjugated Annexin V and propidium iodide (PI) using the Annexin V‐FITC kit (Miltenyi Biotech). Data were collected by flow cytometry. Cell cycle analysis was performed as previously described (13).

### 2.6 Western blotting analysis

Cells were lysed in modified RIPA buffer (10 mM Tris‐HCl, pH 7.5, 150 mM NaCl, 1 mM EDTA, 1% Igepal). Protein samples were subjected to electrophoresis on Nupage 4–12% polyacrylamide gel (Life Technologies), and then transferred onto a nitrocellulose membrane (GE Healthcare). Antibodies were: anti‐Myc (#5605; Cell Signaling Technology), and anti‐GAPDH (sc‐47724; Santa‐Cruz Biotechnology, Dallas, TX, USA).

### 2.7 Statistical analysis

Statistical analysis was performed by the two‐tailed unpaired Student’s *t*-test using the GraphPad Prism® software package. Statistical significance was determined by *p* < 0.05.

## 3 Results

### 3.1 IBTK silencing enhances the pro-apoptotic activity of rituximab in NHL cells *in vitro*

Rituximab stands as one of the most frequently utilized chemotherapeutic drugs in patients with aggressive lymphomas, particularly non-Hodgkin’s lymphoma (NHL) (14, 15). Currently, the foremost frontline therapy for NHL involves the combination of Rituximab with chemotherapy (cyclophosphamide, doxorubicin, vincristine, and prednisone), commonly referred to as R-CHOP (16). Nonetheless, this clinical standard underscores the necessity for the development of novel therapeutic approaches.

Recognizing that the acquisition of rituximab resistance often leads to chemotherapy failure in NHL patients (17), we conducted an investigation into the effects of combining Rituximab with IBTK silencing on cell viability. For our *in vitro* experiments, we employed the Ramos cell line as a model of NHL cells due to their relative resistance to rituximab therapy (17).

**Figure 1:**
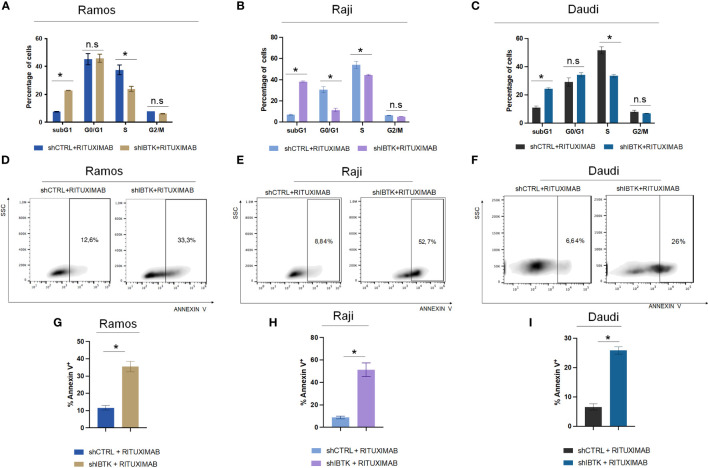
IBTK silencing strongly enhances the pro-apoptotic activity of Rituximab on a panel of B-lymphoma cells *in vitro*. Ramos, Raji and Daudi cells (1x10^6^) were transduced with lentiviral particles (500 ng of p24) expressing short hairpins controls (shCTRL) or directed against IBTK mRNA (shIBTK). Next, cells were treated with or without Rituximab (10 µg/mL) for 48-hours. **(A–C)** shCTRL or shIBTK cells were labeled, fixed then stained with PI/RNase staining solution, and cell cycle analysis was performed by flow cytometry. The phases of cell cycle were evaluated by using the Watson pragmatic model. Values (mean ± SE, n = 3 for each group of samples) are shown. The bar indicates a statistically significant difference according Student’s t test. **(D–F)** Representative density plot of Annexin V binding assay of *in vitro* cultured shCTRL or shIBTK cells treated with Rituximab or left untreated. **(G–I)** Bar diagrams showing the quantification of apoptotic cells by Annexin V binding assay. Values (mean ± SE, n = 3 for each group of samples) are shown. The bar indicates a statistically significant difference according Student’s *t* test. Statistically significant difference was calculated according to Student’s *t* test and represented as asterisk for *p* value < 0.05. n.s. is for not statistically significant.

To achieve this, we transduced Ramos cells with lentiviral particles carrying short hairpin RNA for IBTK silencing (SHI) or a control short hairpin (SHC), followed by treatment with rituximab (10 μg/mL). Remarkably, our observations revealed that IBTK silencing in the presence of rituximab (10 μg/mL) substantially increased the subG1 phase (from 7.65% to 22.8%, p value < 0.005) and reduced the S phase (from 37.5% to 23.9%, p value < 0.05) (**Figure 1A**). These findings indicate a synergistic effect between IBTK depletion and Rituximab treatment on cell viability.

To corroborate the obtained results, we analyzed the cell cycle of NHL cell lines, Raji and Daudi in presence or absence of IBTK silencing treated with rituximab (10 μg/mL). In Raji cell line, we found that the combination of IBTK silencing and Rituximab strongly increased the subG1 phase (from 6.95% to 38.3%, p value < 0.05) along with a reduction of the percentage of cells in G0/G1 phase (from 30.9% to 12.25%, p value < 0.05), in S phase (from 56.55% to 44.75%, p value < 0.05) (**Figure 1B**). Consistent with the results obtained in the other NHL cell lines, in Daudi cell, we observed that the synergistic effect of IBTK silencing and Rituximab caused an increase of percentage of cell in subG1 phase (from 11.1% to 24.4%, p value < 0.005) along with a reduction of the percentage of cells in S phase (from 51.7% to 33.57%, p value < 0.05), (**Figure 1C**).

Subsequently, we investigated whether the synergistic effect resulting from the depletion of IBTK in combination with Rituximab treatment was attributed to the induction of apoptosis. Employing the Annexin V assay, we observed that the combination of IBTK silencing and Rituximab significantly increased the percentage of Annexin V-positive cells to 35.83%, whereas the apoptotic rate of Ramos cells treated with Rituximab alone was 11.65% (**Figures 1D, 1G**). Consistent with this result, the analysis of apoptosis by Annexin V assay, in Raji and Daudi cell lines silenced or not for IBTK and treated with Rituximab (10 mg/mL) showed an increase of the percentage of Annexin V-positive cells from 8.78% (SHC) to 51,23% (SHI) for Raji (**Figures 1E, H**) and from 6.61% (SHC) to 25.87% (SHI) for Daudi cells (**Figures 1F, I**). These results indicate consistency in the trends observed with the Ramos cell line, suggesting a broader applicability of our findings across different NHL cell types (**Figure 1**).

**Figure 2:**
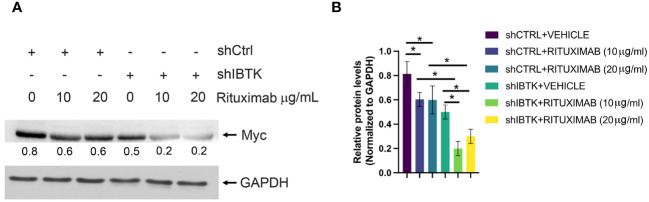
IBTK silencing with Rituximab treatment significatively reduces Myc protein expression on B-lymphoma cells *in vitro*. **(A)** Whole cell extracts (30 µg) from shCTRL or shIBTK Ramos, cells treated with Rituximab (10 µg/mL) or left untreated for 48 hours were separated by 12% SDS–PAGE and analyzed by western blotting using anti-MYC and anti-GAPDH antibodies. **(B)** Densitometric values of the Myc protein bands were normalized to GAPDH bands. Mean values ± SE are shown for three independent experiments. Statistically significant difference was calculated according to Student’s *t* test and represented as asterisk for *p* value < 0.05.

Given previous studies indicating MYC’s involvement in regulating cell cycle inhibitors during the early subG1 phase, influencing the transition from the G1 to the S phase (18), we sought to determine whether the combined effect of IBTK silencing and Rituximab treatment affected MYC protein levels. As anticipated, our findings demonstrated a more pronounced reduction in MYC protein levels in Ramos cells when both IBTK depletion and Rituximab treatment were employed, compared to either IBTK depletion or Rituximab treatment alone (**Figures 2A, B**).

Collectively, these data suggest that the combination of IBTK silencing and Rituximab treatment induces a cell cycle block at the G1-S phase, which is associated with an increase in apoptotic rates, likely attributable to the reduction in MYC protein content (**Figures 1A-C**, **2A, B**).

### 3.2 Combined treatment of rituximab and IBTK silencing decreases lymphoma growth *in vivo*

As IBTK silencing demonstrated inhibitory effects on B-lymphoma growth *in vitro*, we sought to investigate whether the loss of IBTK could exert anti-tumor effects in the treatment of lymphomas in an *in vivo* setting.

To address this, we employed the transplantability of Eµ-myc lymphomas, a preclinical mouse model of NHL (19), into immunocompetent C57BL/6 background strain mice. Specifically, we subcutaneously injected lymphoma cells (1x10^6^ cells) derived from *Ibtk^+/+^ Eµ-myc* and *Ibtk* ^-/-^ *Eµ-myc* lymphomas into C57BL/6 wild-type mice. When each tumor volume reached approximately 100 mm^3^, which occurred about three days later, we administered vehicle or Rituximab intraperitoneally at a dose of 10 mg/kg.

**Figure 3:**
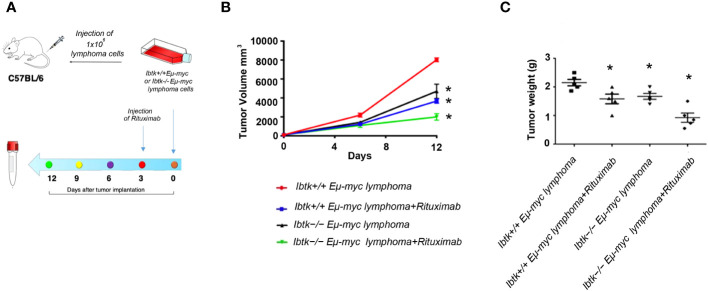
Effects of IBTK silencing combined with Rituximab treatment on *in vivo* model of B-lymphomas. **(A)** Workflow of the experimental design of *in vivo* analysis **(B)** Tumor growth curves of lymphoma cells (1x10^6^), deriving from *Ibtk^+/+^Emu-myc* or *Ibtk^-/-^Emu-myc* lymphomas, subcutaneously injected into C57BL/6 wild type mice. When each tumor volume reached about 100 mm^3^, approximately three days after tumor engraftments, recipient mice were intraperitoneally treated with rituximab (10 mg/kg) or vehicle for indicated time. Values (mean ± SE, n = 5 for each group of samples) are shown. The asterisk indicates a statistically significant difference according Student’s *t* test (*p* < 0.01). **(C)** Weights of tumors deriving from subcutaneous injection of *Ibtk^+/+^Emu-myc* or *Ibtk-^/-^Emu-myc* lymphoma cells (1x10^6^) after treatment with Rituximab (10 mg/kg) or vehicle for six days. Values (mean ± SE, n = 5 for each group of samples) are shown. The bar indicates a statistically significant difference according Student’s *t* test. Statistically significant difference was calculated according to Student’s *t* test and represented as asterisk for *p* value < 0.05.

Notably, we observed a significant reduction in both tumor volume and weight for tumors derived from *Ibtk* ^-^ *^/-^ Eµ-myc* in comparison to those arising from *Ibtk^+/+^ Eµ-myc*. Furthermore, the combined absence of IBTK and Rituximab treatment demonstrated a synergistic effect in reducing both tumor volume and weight (*p* value < 0.001) (**Figures 3A–C**).

## 4 Discussion

The treatment of aggressive lymphomas, particularly non-Hodgkin’s lymphoma (NHL), represents a significant clinical challenge due to the emergence of resistance to Rituximab, a commonly employed chemotherapeutic agent (14, 15). The current standard of care for NHL involves the combination of Rituximab with chemotherapy, known as R-CHOP (16). Nevertheless, the development of rituximab resistance frequently leads to chemotherapy failure in NHL patients (17). To address this challenge, we embarked on a comprehensive investigation into the potential therapeutic benefits of combining Rituximab with IBTK (Inhibitor of Bruton Tyrosine Kinase) silencing, focusing on the enhancement of pro-apoptotic activity. Our *in vitro* results demonstrated that IBTK silencing in combination with Rituximab significantly increased the subG1 phase of cell cycle distribution and reduced the S phase. This shift towards a higher proportion of cells in the subG1 phase signifies an increase in apoptotic cells (17). The synergistic effect observed in our experiments underscores the potential of IBTK silencing to enhance the pro-apoptotic activity of Rituximab. Numerous studies have established that MYC deregulation is one of the most important events for aggressive B-lymphoma malignant transformation (20, 21). Overexpression of MYC is associated with high growth rates *in vivo* and in cell culture experiments, driving quiescent cells into the cell cycle (22). In contrast, low levels of MYC are associated with nondividing and differentiated cells (23). Previous studies showed that IBTK is a potential transcriptional target of MYC in aggressive MYC-driven B cell lymphomas (4, 24).

The observation of IBTK silencing-dependent apoptosis of cancerous B cells was consistent with previous observations in mouse embryonic fibroblasts (24), in DeFew and MEC-1 human B cell lines (3), and mouse pre-cancerous B-lymphoma cells (4). Interestingly, in our recent work, we identified as a direct interactor of IBTK, GSK3β (6), an essential component of the β-catenin destruction complex (25). β-catenin pathway is reported to be involved in the progression of many types of cancers, such as leukemia (26–28), myeloma (28, 29), and several subtypes of lymphoma (30, 31). Our study showed that down-regulation of IBTK could significantly inactivate β-catenin signaling through the stabilization of GSK3β which promotes β-catenin degradation, thus preventing the nuclear accumulation of β-catenin as well as the transcriptional activation of its target genes MYC, CCDN1, and CD44 (6).

Moving from our promising *in vitro* results to an *in vivo* setting, we utilized a preclinical mouse model of NHL to investigate the potential anti-tumor effects of IBTK loss. Our results demonstrate a significant reduction in both tumor volume and weight when IBTK was absent in conjunction with rituximab treatment. The synergistic reduction in tumor growth, highlighted in our study, is a noteworthy finding and underscores the potential clinical significance of combining IBTK silencing with Rituximab.

Long-term exposure of the tumor to certain drugs can lead to drug resistance and multi-drug combination is a promising approach to overcome drug resistance (32, 33). Intriguingly, we found that the combination of IBTK silencing with Rituximab treatment induces a cycle block at the G1-S phase associated with the reduction of MYC protein content and concomitantly to an increase of apoptotic rate. In addition, we found that IBTK loss and Rituximab treatment act synergistically in reducing tumor volume and weight in the Eµ-myc mouse model of non-Hodgkin’s lymphoma (NHL) (34). While our results offer promising insights, several limitations warrant consideration. First of all, our study predominantly focused on B-NHL cell lines and only one mouse model of B-NHL, not fully capturing the heterogeneity of NHL in clinical populations. We assume that we will also extend to test the combined approach in more complex and immunocompetent preclinical models, such as humanized mice. The consistency of the trends observed in our models (Ramos, Raji and Daudi cell lines) suggests a broader applicability of our findings across different NHL cell types (**Figure 1**). Of course, further investigations are needed to validate the translational potential of our findings. Our study presents compelling evidence that the combination of IBTK silencing with Rituximab enhances pro-apoptotic activity in NHL cells, both *in vitro* and *in vivo*. This holds great promise for addressing rituximab resistance and advancing the development of innovative therapeutic regimens for aggressive lymphomas. Future studies should aim to further elucidate the underlying mechanisms and expand the clinical applicability of this approach. Primary concern will be a consideration of regulatory approval pathways for precision medicine approaches, including early-phase studies in cohorts selected based on the IBTK profile. In addition, the evaluation of combinations with other targeted therapies, such as inhibitors of the PI3K/AKT pathway or CD20 signaling, and the assessment of the synergistic impact with immunotherapy, given its growing relevance in lymphoma, will be integral to our ongoing investigations.

## Data availability statement

The raw data supporting the conclusions of this article will be made available by the authors, without undue reservation.

## Ethics statement

The animal study protocol was approved by the Bioethical Committee of the University Magna Graecia of Catanzaro. The animal experiments were carried out in accordance with the protocol n.794/2016‐PR, approved by the Italian Ministry of Health. The studies were conducted in accordance with the local legislation and institutional requirements. Written informed consent was obtained from the owners for the participation of their animals in this study.

## Author contributions

EV: Conceptualization, Data curation, Investigation, Methodology, Writing – original draft, Validation. RM: Methodology, Writing – original draft. SM: Data curation, Methodology, Writing – review & editing. CCan: Methodology, Writing – original draft. CCai: Methodology, Writing – review & editing. AA: Formal analysis, Writing – review & editing. MRR: Methodology, Writing – review & editing. MS: Data curation, Methodology, Writing – review & editing. GS: Data curation, Writing - review & editing. CP: Data curation, Writing – review & editing. EI: Data curation, Writing – review & editing. MM: Data curation, Formal analysis, Writing – review & editing. IQ: Conceptualization, Supervision, Validation, Visualization, Writing – review & editing. GF: Conceptualization, Data curation, Formal analysis, Funding acquisition, Investigation, Methodology, Resources, Supervision, Writing – original draft, Writing – review & editing.
